# Biochar addition enhances remediation efficiency and rapeseed yield in copper-contaminated soil

**DOI:** 10.3389/fpls.2024.1481732

**Published:** 2024-10-17

**Authors:** Ziwei Sheng, Tao Luo, Linjie Wang, Min Chen, Bingbing Ma, Lijun Liu, Bo Wang, Jie Kuai, Jing Wang, Jie Zhao, Zhenghua Xu, Guangsheng Zhou

**Affiliations:** Ministry of Agriculture (MOA) Key Laboratory of Crop Ecophysiology and Farming System in the Middle Reaches of the Yangtze River, College of Plant Science and Technology, Huazhong Agricultural University, Wuhan, Hubei, China

**Keywords:** copper contamination, oxidative stress, cell wall, rapeseed, soil amendment

## Abstract

**Introduction:**

Soil contamination with copper (Cu) threatens ecological security and human health. Rapeseed demonstrates potential in remediating copper-contaminated soil, and biochar-assisted phytoremediation is increasingly being employed to improve remediation efficiency. However, the combined application of them has not been thoroughly studied in terms of the synergistic effects and the mechanisms of their interaction.

**Methods:**

In this regard, this study conducted a pot experiment to evaluate biochar-assisted remediation under Cu-contaminated soil with varying biochar application rates; Furthermore, the plant physiological mechanism and soil physicochemical properties involved in the biocharrapeseed system was explored.

**Results:**

Our results showed that the exchangeable pool of copper in soil decreased by 10.0% and 12.3% with adding 5% biochar (BC1) and 10% biochar (BC2) relative to control (BC0), respectively, prior to rapeseed cultivation. The rapeseed cultivation for one season further reclaimed 4.9%, 9.0%, and 13.6% of the available copper in this soil by root extraction under the BC0, BC1, and BC2 treatments, respectively. The overall copper concentration in plants decreased by 23.7% under BC2 and 13.3% under BC1 compared to BC0. However, the plant’s dry biomass at BC1 and BC2 treatments increased by 1.7-fold and 2.7-fold relative to BC0, which offset the negative impact of the decreased copper concentration on phytoremediation. Physiological analysis showed adding 10% biochar decreased the MDA content by 36% in the leaf and 49% in the root, compared to BC0. The transmission electron microscopy for cell wall ultrastructure in root tips showed that biochar addition in Cu-contaminated soil increased the mechanical strength of the celL wall, explicitly increasing the thickness of the secondary cell wall. Further cell wall components analysis revealed a remarkable increment of the pectin content in BC2 relative to BC0, increased by 56% in the leaf and 99% in the root, respectively. Additionally, 10% biochar application led to a roughly 2-fold increase in seed yield via ameliorating the soil physicochemical properties and increasing the rapeseed growth.

**Discussion:**

These findings offer insights into synergistic rapeseed-biochar use for Cu-contaminated soil remediation.

## 1 Introduction

Environmental pollution caused by heavy metals is increasingly raising concerns over the potential effects on ecological safety and human health. Rapid industrialization and irrational anthropogenic activity lead to the generation and release of a large amount of heavy metals into the soil profile. According to the nationwide extensive survey in China, the total area of arable land polluted with heavy metals has reached 20 million hectares, accounting for approximately 16.1% of the entire arable land (Sodango et al., 2018). Copper is abundantly present in copper mines as chalcocite (Cu_2_S), covellite (CuS) and malachite (Cu_2_(OH)_2_CO_3_) (Adams et al., 2008). When exposed to oxygen and water, these compounds become unstable and release their monovalent cation into the soil. Soil contamination with copper is the main heavy metal pollution besides arsenic and cadmium in China (Huang et al., 2018). Copper is an essential micronutrient for living organisms. However, elevated copper concentration in soil substantially impacts plant productivity and survival (Kumar et al., 2021). It stimulated the excessive release of reactive oxygen species, which have deleterious effects on cellular membranes, molecular functions and biological processes (Hartley-Whitaker et al., 2001).

It is imperative to strike a balance between environmental remediation and economic benefit concerning the agricultural utilization of contaminated soils. Conventional techniques to remediate heavy metals from contaminated soils are based on physical, chemical, and biological methods and integrated approaches (Azhar et al., 2022). In general, these soil remediation methods employ extraction or immobilization mechanisms to reduce the toxicity level of heavy metals (Liu et al., 2018). Physiochemical technologies may be highly efficient but are costly and environmentally destructive (Khalid et al., 2017). Phytoremediation is to grow plants in contaminated soils to remove heavy metals or stabilize them into harmless status (Rascio and Navari-Izzo, 2011). This plant-based approach is perceived as operationally simple, eco-friendly, and solar-driven technology with good public acceptance (Ali et al., 2013). However, plant remediation potential is limited in terms of the slow growth in toxic environments and the long time required for soil clean-up (Kozminska et al., 2018).

Biochar-enhanced phytoremediation is an emerging strategy to achieve a synergistic and effective effort to remediate heavy metal-contaminated soils (Selvi et al., 2019). Biochar is a porous carbonaceous material produced from the anaerobic pyrolysis of biomass (Haeldermans et al., 2020). The specific thermochemical procedure confers biochar the characteristics of high porosity, large surface area, and diverse functional groups (Liang et al., 2021). The application of biochar as an adsorbent in contaminated soil remediation has recently attracted increased attention to immobilize heavy metals in the environment, thus reducing the bioavailability and biotoxicity of heavy metals to the organisms (He et al., 2019; Liu et al., 2022). A meta-analysis from 227 peer-reviewed articles revealed that biochar application decrease in Cd level ranged from 24.9% to 45.0%, and this variation was mainly attributed to the feedstock, application rate and pH of biochar and soil physicochemical properties (Duan et al., 2023). A field experiment also reported that phytoremediation-biochar synergy decreased the heavy metal concentrations in the rhizosphere by 30%-40% compared with the control (Jun et al., 2020).

Rapeseed (*Brassica napus* L.) has often been highlighted as a candidate to accumulate significant amounts of Cu in its tissues when grown in copper mine-impacted soils (Gasco et al., 2019; Munir et al., 2020). Although rapeseed has a considerable growth rate and strong adaptability to adverse environments, copper concentration at high levels largely restricted its growth and remediation potential (Feigl et al., 2013). In this scenario, the main objective of this study was to evaluate the remediation efficiency and seed yield of rapeseed under Cu-contaminated soil through biochar-assisted phytoremediation. Two different doses of rice straw biochar were applied in the Cu-contaminated soil for rapeseed growth. We investigated the copper bioaccumulation and translocation in various tissues of rapeseed plants. In addition, the physiological responses of rapeseed toward excessive copper stress were measured under different amounts of biochar application. More specially, we monitored the pre-sowing and post-harvesting bioavailable copper concentration in tested soils. The present study aims to provide valuable insights into the feasibility of applying phytoremediation-biochar synergy to remediate Cu-contaminated soil for ecological benefits and to produce edible or biofuel oil for economic benefits.

## 2 Material and methods

### 2.1 Soil and biochar collection

**Figure 1:**
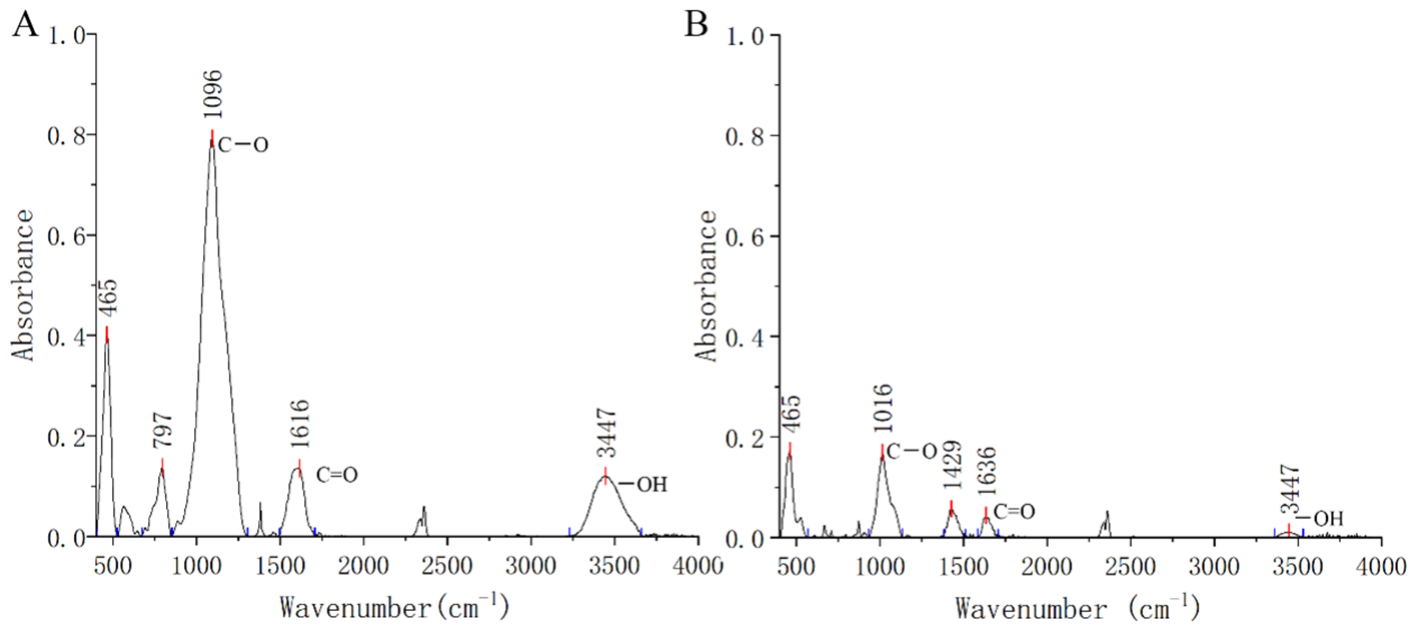
Fourier transform infrared (FTIR) spectrum of rice straw biochar **(A)** and soil contaminated soil **(B)**. The stronger peaks at about 3440 cm^−1^, 1616 cm^−1^, and 1096 cm^−1^ were connected to the presence of -OH, C=O, and C-O, respectively.

The copper-contaminated soil was collected from the surface (0–20 cm) of copper mining tailings in Baisha Village, Daye County, Hubei Province, China (115.20′E, 29.85′N). The bulk soil sample was air-dried at room temperature and thoroughly mixed after passing through a 5-mm nylon sieve. The rice straw biochar (RSB, prepared at 300°C) was obtained from an organic biochar manufacturer in Hubei, China. Transform Infrared Spectroscopy (FITR) analysis was conducted to illustrate the contents of functional groups in biochar and Cu-contaminated soil. The samples were dried in the oven at 45°C for 24 h and then quantitively mixed with KBr powder (1 mg sample with 100 mg KBr) to make a pellet for FITR analysis. The spectra were recorded as an average of 32 scans using a VERTEX70 FTIR spectrometer (Bruker, Hamburg, Germany) with a wavelength resolution of 4 cm^−1^ in the 400–4000 cm^−1^ range. The FITR results are shown in **Figure 1**. The base physicochemical features of the copper-contaminated soil and biochar are presented in **Table 1**.

**Table 1:** Basic physicochemical properties of copper-contaminated soil and rice straw biochar used in the present study.

Properties	Copper contaminated soil	Rice straw biochar
pH	7.08 ± 0.02	8.25 ± 0.09
CEC (cmol kg^−1^)	6.28 ± 0.47	16.1 ± 2.04
Total copper concentration (mg kg^-1^)	1055.4 ± 63.9	–
Organic matter (g kg^-1^)	2.78 ± 0.28	–
C (%)	–	58.6 ± 4.2
N (g kg^-1^)	0.17 ± 0.02	3.94 ± 0.16
P (g kg^-1^)	0.32 ± 0.05	7.80 ± 0.63
K (g kg^-1^)	2.59 ± 0.14	27.6 ± 4.35

The values are expressed as mean ± standard error for n = 3 replications and “-” indicates value not available. CEC, cation exchange capacity; C, soil carbon content; N, soil nitrogen content; P, soil phosphorus content; K, soil potassium content.

### 2.2 Pot experiment designation and plant cultivation

A pot experiment was conducted from Oct 2022 to May 2023 in the glasshouse at Huazhong Agricultural University, Wuhan, China (114°21′E, 30°01′N). Each plastic pot (height of 24 cm and bottom diameter of 33 cm, respectively) was filled with 10 kg of Cu-contaminated soil on a dry weight basis. The soil was then homogeneously amended with RSB at 0, 5% and 10% (w/w) on a dry soil basis and incubated for 60 days at 70% soil water holding capacity. The pots were arranged in a completely randomized design (CRD) with six replicates. The rapeseed (*Brassica napus* L.) cv. ‘H2009’ was used in the present study for phytoremediation purposes, which showed considerable resistance to copper stress. Each pot was sown with ten intact seeds and manually thinned into two uniform plants per pot at the seedling emergence stage. Before sowing, each pot was mixed homogeneously with 2.18 g urea, 10.0 g calcium superphosphate, and 3.0 g potassium chloride as base fertilizer. Another 2.18 g urea was applied as topdressing to each pot at the budding stage. The soil moisture content in pots was monitored by Fieldscout TDR300 soil moisture meter (Spectrum Technologies Inc. USA) in 2-3 days intervals and maintained at 60-80% soil water holding capacity during the growing season. The ambient mean daily temperature during the rapeseed growth period initially reached 30°C in Oct, then fluctuated downward to –0.4°C by Jan, and subsequently rose with fluctuations to 25°C by May. The weeds in the pots were manually removed, and pesticides were used at specific and required times to protect plants from diseases and insect damage.

### 2.3 Oxidative levels and antioxidant system

The fresh leaf and root of rapeseed plant were sampled 90 days after the sowing (vegetative stage) to determine the levels of H_2_O_2_, O_2_^-^, malondialdehyde (MDA), proline, ascorbate peroxidase (APX; EC 1.11.1.11), catalase (CAT; EC 1.11.1.6), superoxide dismutase (SOD; EC 1.15.1.1) and peroxidase (POD; EC 1.11.1.7). The fresh samples were rinsed twice in distilled water, blotted dry, immediately frozen in liquid nitrogen, and stored in a freezer at a low temperature (-80°C) for subsequent analysis. The MDA was extracted with 5% trichloroacetic acid and reacted with 0.67% (W/V) thiobarbituric acid. The absorbance of the adduct recorded at 450 nm, 532 nm, and 600 nm was used to calculate the MDA content (Janero, 1990). Proline was extracted by 3% 5-sulfosalicylic acid, and the ninhydrin-based colorimetric assay was employed to quantify the proline content by measuring the absorbance at 520 nm with a spectrophotometer (Ábrahám et al., 2010). APX, CAT, SOD, and POD activities were determined by the Assay Kits (Jiangsu Jingmei Biotechnology Co., Ltd, China) following the standard protocol, respectively. Briefly, 0.1 g of the fresh sample was accurately weighed and placed into a centrifuge tube, followed by the addition of 0.9 mL phosphate buffer (pH 7.0-7.2). The mixture was centrifuged at 9,300 ×g for 20 minutes at -4°C to obtain the crude enzyme extract, which was then used for enzyme activity measurements. Histochemical localization was carried out to detect H_2_O_2_ and O_2_^-^ by using the 3,3-diaminobenzidine (DAB) and nitroblue tetrazolium (NBT) staining methods, respectively (Wang et al., 2007).

### 2.4 Root morphology and cell ultrastructure

The roots were carefully excavated from pots and hand-washed using tap water. Following that, the sampled roots were spread in a rectangular and transparent acrylic tray filled with distilled water and scanned in grey-scale mode at 300 dpi using Epson V800 scanner. The obtained root images were then analyzed using WinRHIZO 2017 Pro software (Regent Instruments, Canada) to determine root morphology such as total root length (TRL), root surface area (RSA), root volume (RV), root diameter (RD) and root tips (RT). Root activity was analyzed by the triphenyl tetrazolium chloride (TTC) method (Zhang et al., 2013). In brief, a 0.2 g fresh root sample was immersed in 10 mL of equally mixed solution of 0.4 g L^-1^ TTC and phosphate buffer (pH=7) and kept in the dark at 37°C for 2 h. Subsequently, 2 mL of 1 mol L^-1^ H_2_SO_4_ was added to terminate the chemical reaction within the root. The root was dried with absorbent paper and then transferred to a 2 mL centrifuge tube, adding 1 mL of ethyl acetate to crush at 70 Hz for 10 minutes. The red homogenate was washed into the volumetric flask to reach 10 mL using ethyl acetate, and the absorbance of the extract was recorded at 485 nm by spectrophotometer. The root activity was expressed as TTC reduction intensity. For ultrastructural observation, the root tips of rapeseed were cut into pieces of approximately 3 mm^2^, fixed with 2.5% glutaraldehyde in 0.1 M sodium cacodylate buffer at 4°C for 2h and postfixed in 1% osmium tetroxide for 2h, followed by dehydration in gradient ethanol (30%, 50%, 70%, 90%, and 100%). The dehydrated sample was permeated in the mixture of acetone and Epon812 (1:1, V/V) for 12h, then embedded in pure Epon812 for 12 h. The embedded blocks were polymerized at 60°C for 48 h and sectioned with UC7 ultramicrotome (Leica, Germany), double-stained with uranyl acetate and lead citrate for 15 mins. Transmission electron microscopy (TEM) observations of root tip cells were carried out by using a Tecnai G^2^ 20 S-TWIN microscope (Tecnai G^2^ F20 S-TWIN, FEI, USA).

### 2.5 Cell wall components determination

The pectin, hemicellulose, and cellulose in the cell wall of the root and leaf were determined as described previously with minor modifications (Li et al., 2017; Zhong and Lauchli, 1993). The sample powder was successively washed twice with potassium phosphate buffer (pH 7.0), chloroform-methanol, and DMSO-water. The homogenates of each wash were centrifuged for 5 mins at 1800 ×g, and the final collected precipitation was considered crude cell wall. The pectin fraction was obtained by extracting twice the dry crude wall material with 0.5% (w/v) ammonium oxalate, heating for 1 h in a boiling water bath, and pooling the supernatants. The remaining pellets were suspended in 5 ml of 4 M KOH containing 1.0 mg/mL sodium borohydride for 1 h at 25°C and then centrifuged at 1800 ×g for 5 mins. The procedure above was repeated two times to collect the supernatants as KOH extractable hemicelluloses. The remaining pellets were sequentially extracted with TFA as non-KOH-extractable hemicelluloses. The pellets were further extracted with acetic-nitric acids-water for 1 h at 100°C, and the remaining pellets were regarded as crystalline cellulose. Pectin content was expressed by measuring the content of galacturonic acid (Jia et al., 2019). Cellulose content was measured using the anthrone/H_2_SO_4_ method, and hemicellulose content was calculated according to the total hexoses and pentoses detected (Li et al., 2015).

### 2.6 Copper concentration determination in soil and plant

The harvested plants at maturity were separated into root, leaf, stem, pericarp, and seed to determine the copper concentration in plant tissues. The detached leaves from each plant were gathered during the whole growth period. The separated plant organs were oven-dried at 105°C for 30 mins to kill all the metabolism and oven-dried at 75°C to constant weight. The dried samples were ground into powdered form and passed through a 0.1mm sieve. Around 0.5 g sample powder was digested for Cu determination using a CEM MARS 6™ microwave digestion instrument (CEM Corporation, Matthews, NC, USA) through HNO_3_-H_2_O_2_ solution. The final reading for calculating copper concentration was taken from atomic absorption spectrometry (AAS, Agilent 240FS-AA, USA). The pre-sowing and post-harvesting soils in each pot were collected to determine the bioavailable copper concentration in soil profiles. The available copper concentration in soil was extracted by a diethylenetriaminepentaacetic acid (DTPA)–CaCl_2_–triethanolamine (TEA) solution and measured by atomic absorption spectrometry (Farrag et al., 2012).

### 2.7 The plant harvesting, pre-sowing and post-harvesting soil physicochemical properties

At maturity, the pot plants were harvested and air-dried to constant weight, then threshed to obtain the weight of the plant’s dry biomass and seed yield. The soil in each pot was collected pre-sowing and post-harvesting for soil physico-chemical analysis. The soil pH was measured using a pH meter (Mettler Toledo FE28, China) with a soil–water ratio of 1:2.5 (w/v) after shaking for 1h. The soil cation exchange capacity (CEC) was determined based on the cobaltihexamine chloride absorbance method (Aran et al., 2008). Soil aggregates were separated by the wet-sieving method into five size classes (> 2, 2–1, 1–0.25, 0.25–0.053, and < 0.053 mm) (Xue et al., 2019). Briefly, triplicates of 100g soil were submerged in enough deionized water for 10 mins to allow full slaking. After slaking, the soil was transferred to a soil particle structure analyzer with a series of sieves successively reducing mesh diameter (2, 1, 0.25, and 0.053 mm). It was submerged in water and gently shaken for 10 mins with a 4-cm amplitude vertical vibration. After that, the soils retained in each sieve were washed and transferred into the beaker, and all the sizes of aggregates were oven-dried at 60°C to constant weight. The mean weight diameter (MWD, mm) wascalculated using the following equation:


(1) $$\text{MWD}=\sum_{i=1}^{n}W_{i}\times X_{i}$$

wherein $X_{i}$ and $W_{i}$ are the mean diameter and mass proportion of the aggregate fraction i, respectively (Kemper and Rosenau, 1986).

### 2.8 Remediation determination of biochar and rapeseed

After 2 months’ equilibrium with or without biochar addition in Cu-contaminated soil, the remediation efficiency of biochar application for copper ions was calculated before plant cultivation as following Equation 2:


(2) $$\text{Biochar remediation efficiency }\left(\%\right)=\frac{{\text{BC}}_{0}-{\text{BC}}_{i}/\left(1-i\right)}{{\text{BC}}_{0}}*100$$

wherein ${\text{BC}}_{i}$ is the bioavailable copper concentration after biochar application at a rate i (5% and 10% in the present study) and ${\text{BC}}_{0}$ is the bioavailable copper concentration without biochar application in Cu-contaminated soil. For proper estimation of the reduction of the bioavailable copper concentration of biochar treatment compared to control under the same mass pollutant soil, the bioavailable copper concentration in biochar-applied soil was adjusted by dividing by the mass proportion of copper-contaminated soil in the pot. The phytoremediation efficiency of rapeseed was calculated as following Equation 3:


(3) $$\text{Rapeseed phytoremediation efficiency }\left(\%\right)=\frac{C_{initial}-C_{post}}{C_{initial}}*100$$

wherein $C_{initial}$ and $C_{post}$ are the pre-sowing and post-harvesting bioavailable copper concentrations in soil (Tanhan et al., 2007).

### 2.9 Statistical analysis

Variance (ANOVA) analysis for the treatment effects was conducted using the SPSS program (Version 19.0, SPSS Inc., IL, USA). The Fisher’s protected least significance difference (LSD) test with *P* < 0.05 was used for multiple comparisons between treatment means. The graphical presentation was carried out using R 4.1.1 software (R Core Team, 2018).

## 3 Results

### 3.1 Plant growth and copper uptake

**Figure 2:**
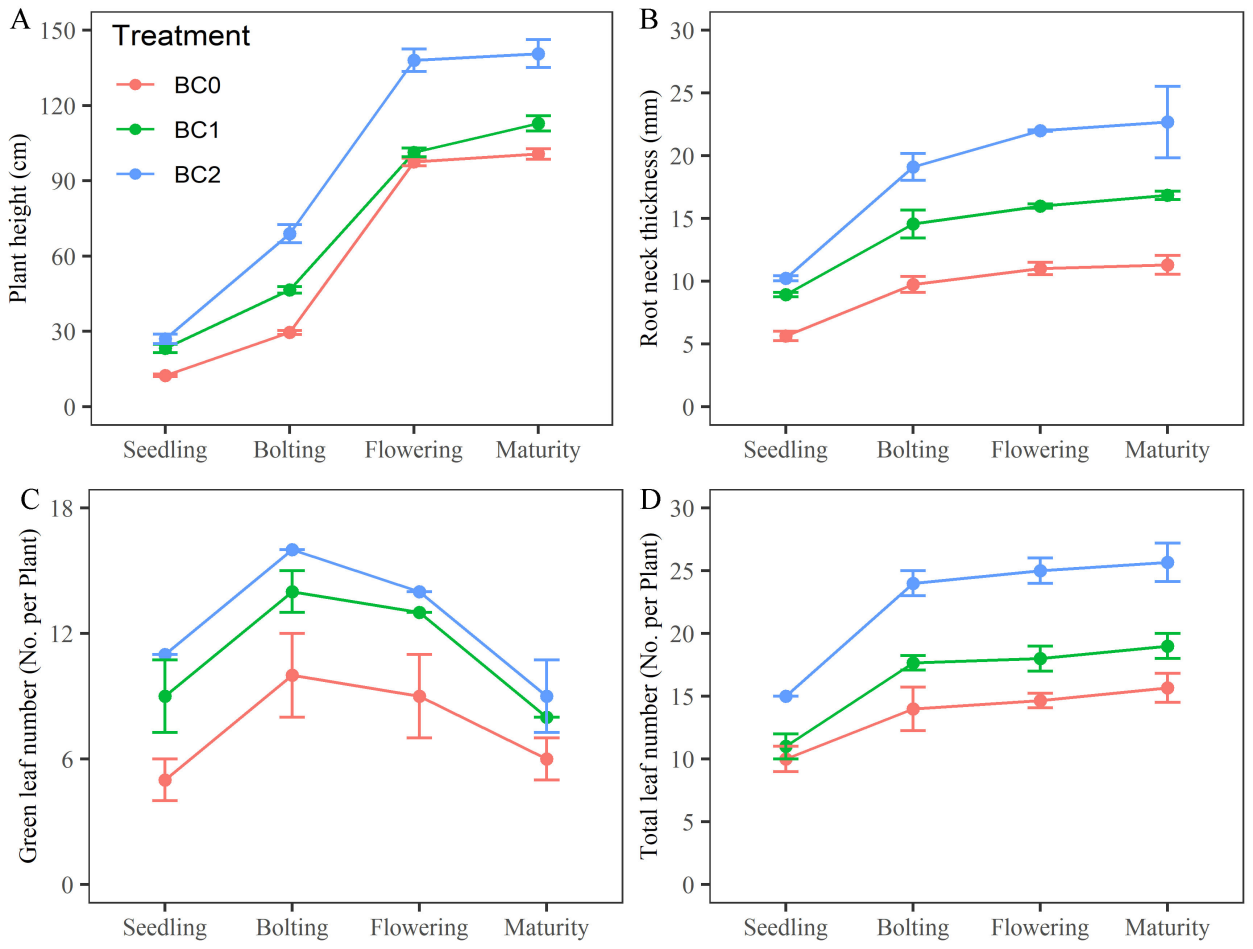
Effects of varying biochar application rates on rapeseed growth in copper-contaminated soil. **(A)** plant height; **(B)** root neck thickness; **(C)** green leaf number; **(D)** total leaf number.

The effects of different biochar application rates on rapeseed growth in copper-contaminated soil are shown in **Figure 2**. Compared to BC0, plant height under BC1 treatment was approximately 1.86, 1.57, 1.04, and 1.12 times greater at the seedling, bolting, flowering, and maturity stages, respectively; under BC2 treatment, plant height increased to 2.16, 2.33, 1.42, and 1.40 times at these stages (**Figure 2A**). Root neck diameter increased to 1.58, 1.49, 1.45, and 1.49 times under BC1 and to 1.82, 1.96, 2.00, and 2.01 times under BC2 compared to BC0 (**Figure 2B**). Additionally, under BC2, green leaf number increased by 2.20, 1.60, 1.56, and 1.50 times across the same growth stages (**Figure 2C**), while total leaf number increased by 1.50, 1.71, 1.70, and 1.64 times, relative to BC0 (**Figure 2D**).

**Figure 3:**
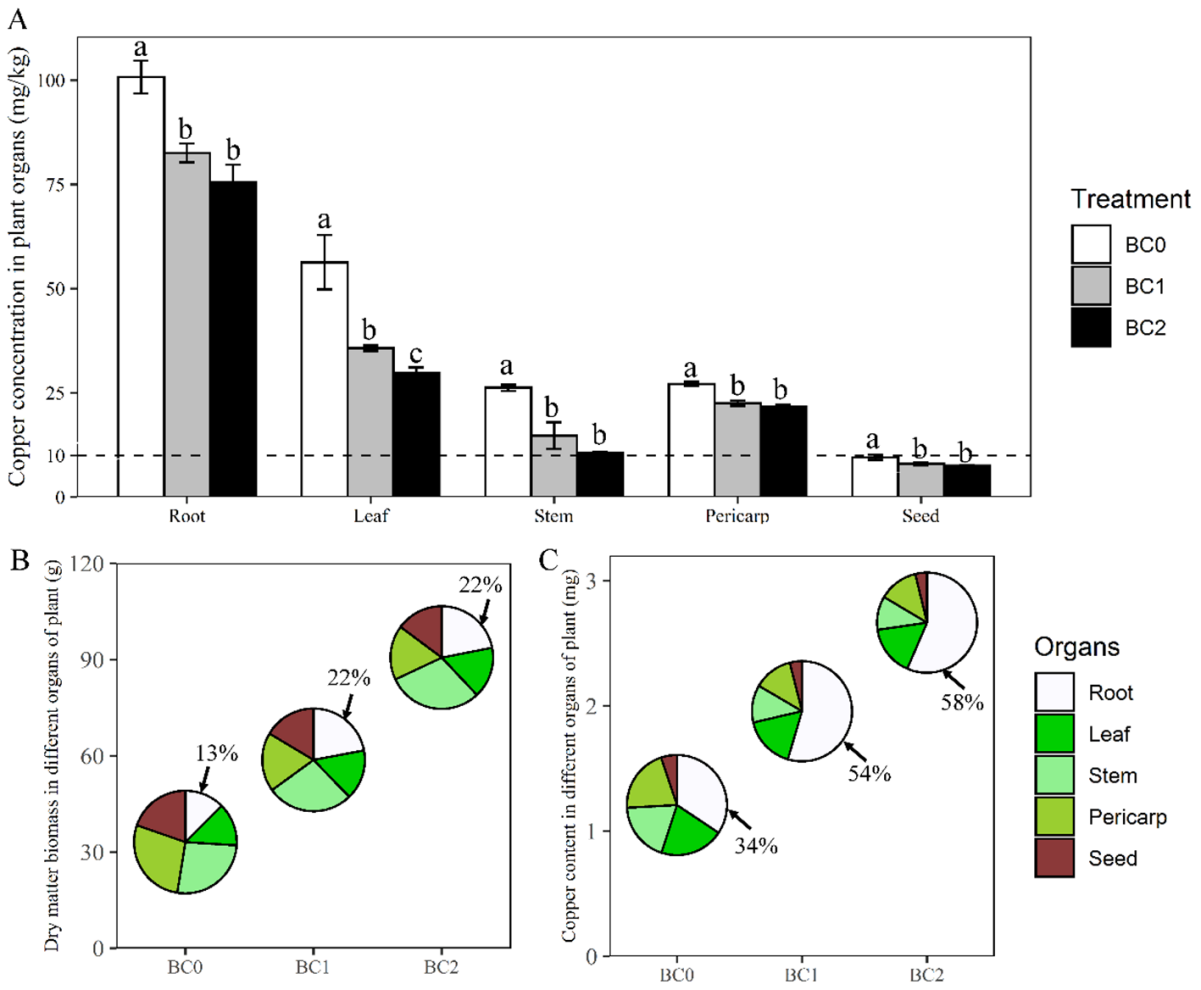
Changes in rapeseed dry biomass and copper uptake under different biochar application rates in Cu-contaminated soil. **(A)** The copper concentration (per gram of dry weight) in various plant organs of rapeseed with the addition of biochar. **(B, C)** The dry biomass and copper uptake amount and their proportions among different plant organs with the addition of biochar, respectively. BC0, BC1 and BC2 indicate the application rates of 0, 5% and 10% of rice straw biochar in Cu-contaminated soil, respectively. The dashed line in panel **(A)** shows the tolerance limit of copper in food provided by standard Chinese GB15199-94. The bars indicate mean ± SD for 3 replications. Different letters on the bar indicate significant differences among treatments at P ≤ 0.05 by LSD.

The plant dry biomass and copper uptake under different application rates of biochar in Cu-contaminated soil are presented in **Figure 3**. Averaging across the biochar treatments, root enriched the highest copper concentration (86.3 mg kg^-1^) among the plant organs, followed by leaf (40.6 mg kg^-1^), pericarp (23.8 mg kg^-1^), stem (17.2 mg kg^-1^) and seed (8.3 mg kg^-1^). Applying biochar in Cu-contaminated soil significantly decreased the copper concentration in the plant organs, compared to the control (**Figure 3A**). The copper concentration in root, leaf, stem, pericarp, and seed decreased by 25%, 60%, 47%, 20% and 21% under BC2 treatment in relative to BC0, respectively. Although copper concentration in different plant organs showed a noticeable decrease with the biochar increase from BC1 to BC2, only the copper concentration in the leaf showed a statistical difference (**Figure 3A**).

The biochar application rate significantly increased plant dry biomass and copper uptake amount (**Figures 3B, C**). The plant total dry biomass at BC1 and BC2 treatments were 1.7-fold and 2.7-fold relative to BC0, respectively; The ratio of root dry weight to plant total biomass were 0.13, 0.22, and 0.22 under the BC0, BC1and BC2 treatments, respectively (**Figure 3B**). The copper uptake amount per plant at BC1 and BC2 treatments were 1.6-fold and 2.2-fold relative to BC0, respectively. The copper uptake in the root accounted for 34%, 54%, and 58% of the total plant-accumulated copper under the BC0, BC1, and BC2 treatments, respectively (**Figure 3C**).

### 3.2 Oxidative stress and antioxidant defense system

**Figure 4:**
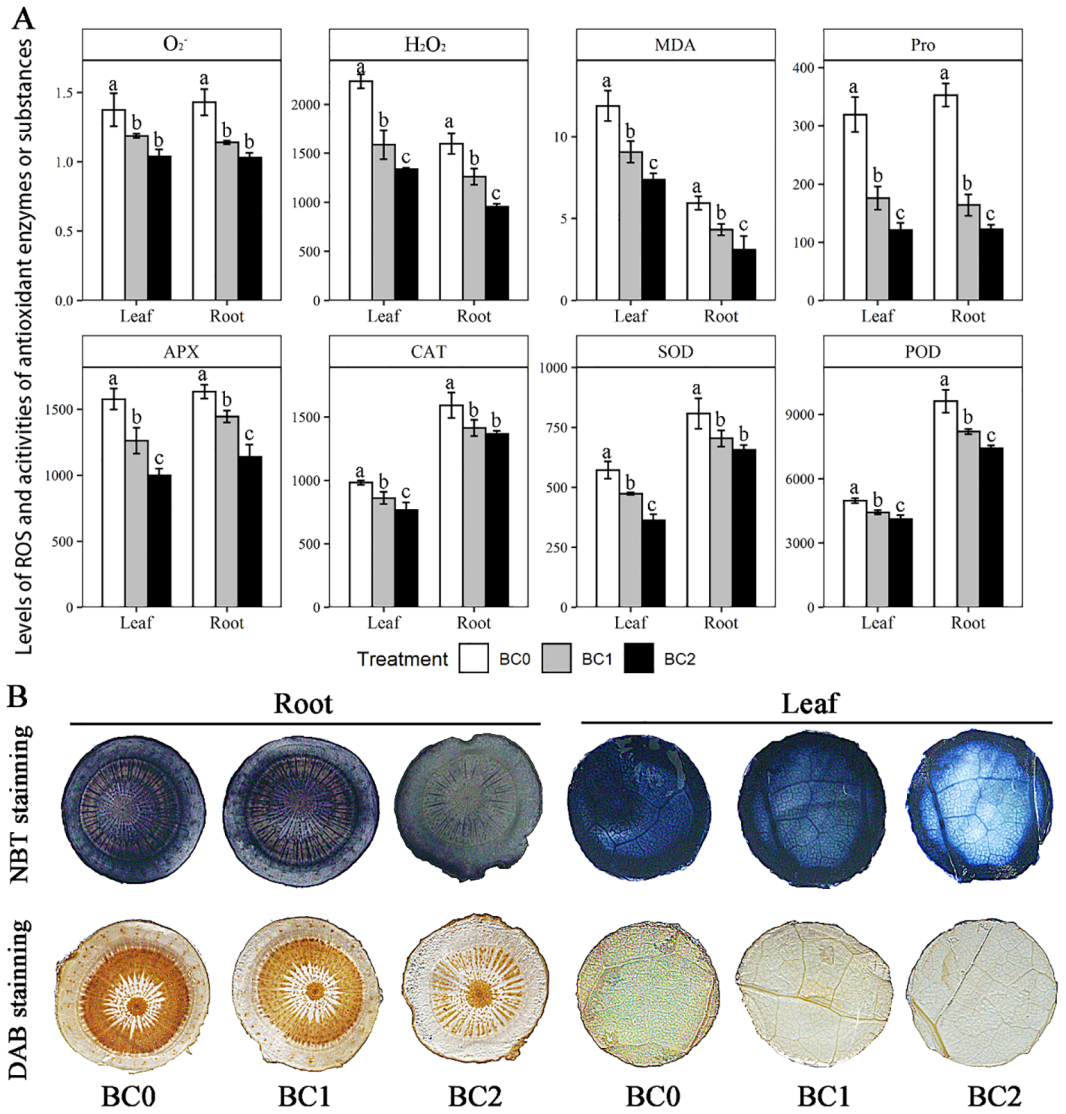
Effects of increasing doses of biochar on ROS and the antioxidant defense system in leaf and root of rapeseed. **(A)** Physicochemical determination of ROS and enzymatic and non-enzymatic antioxidants, including O_2_^-^ (U g^-1^ FW), H_2_O_2_ (mmol g^-1^ FW), MDA (malondialdehyde, mmol g^-1^ FW), Pro (proline, μg g^-1^ FW), AXP (ascorbate peroxidase, U g^-1^ FW), CAT (catalase, U g^-1^ FW), SOD (superoxide dismutase, μg g^-1^ FW) and POD (peroxidase, μg g^-1^ FW). **(B)** Histochemical analyses with 3,3′-diaminobenzidine (DAB) and nitro blue tetrazolium (NBT) for sectioned root and leaf. The bars indicate mean ± SD for 3 replications. Different letters on the bar indicated significant differences among treatments at P ≤ 0.05 by LSD.

Physicochemical analyses showed that the addition of biochar in Cu-contaminate soil notably decreased the ROS levels (O_2_^-^ and H_2_O_2_) and the antioxidant defense system, including enzymatic and non-enzymatic antioxidants (SOD, POD, CAT, APX, and Pro) in both root and shoot (**Figure 4A**). Among the measured physiological variables, the Proline content showed a maximum reduction with the increasing doses of biochar in Cu-contaminated soil, which in BC2 decreased by 66% in leaf and 62% in the root, compared to BC0; Followed by the MDA content, which in BC2 decreased by 36% in leaf and 49% in root, compared to BC0. Averaging across the biochar treatment, the enzymatic activities of SOD, POD, and CAT in the root were 1.6-fold, 1.9-fold, and 1.5-fold of that in the leaf, respectively. Meanwhile, the MDA content in the root was 0.47 times that in the leaf. Furthermore, histochemical analyses with 3,3′-diaminobenzidine (DAB) and nitro blue tetrazolium (NBT) revealed that the staining degree gradually became lighter in root and leaf with the increase of biochar application (**Figure 4B**).

### 3.3 Cell wall components in leaf and root

**Figure 5:**
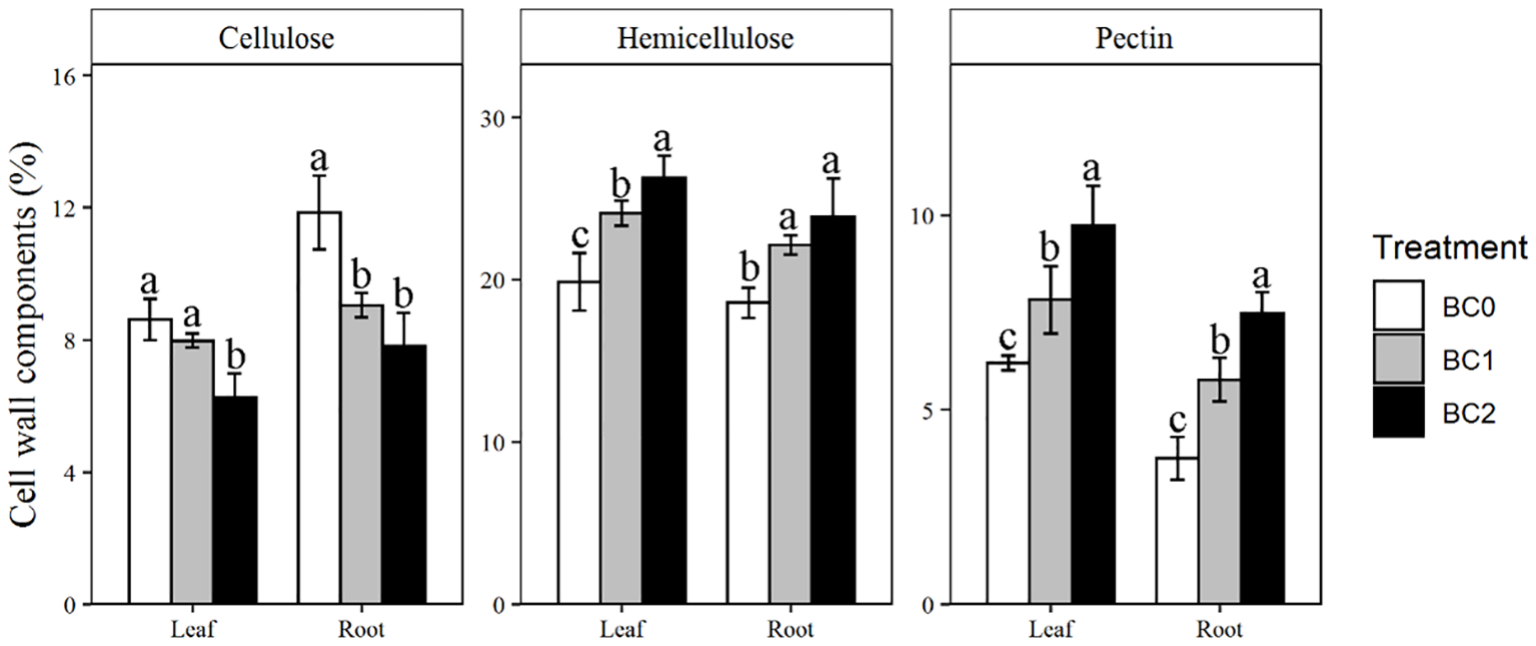
The effects of increasing biochar application rates on cell wall components in root and shoot of rapeseed. The bars indicate mean ± SD for 3 replications. Different letters on the bar indicated significant differences among treatments at P ≤ 0.05 by LSD.

The addition of biochar in Cu-contaminated soil notably affected the cell wall components in the leaf and root (**Figure 5**). Addition of 10% biochar decreased the cellulose content by 27% in the leaf and 34% in the root, respectively, compared to BC0. In contrast, the hemicellulose was increased with the addition of biochar. Compared to BC0, BC2 treatment increased the hemicellulose contents by 32% in the leaf and 28% in the root, respectively. The pectin content in BC2 showed the most remarkable increment among the cell wall components relative to BC0, increased by 56% in the leaf and 99% in the root, respectively.

### 3.4 Root morphology, activity and cell wall ultrastructure

**Figure 6:**
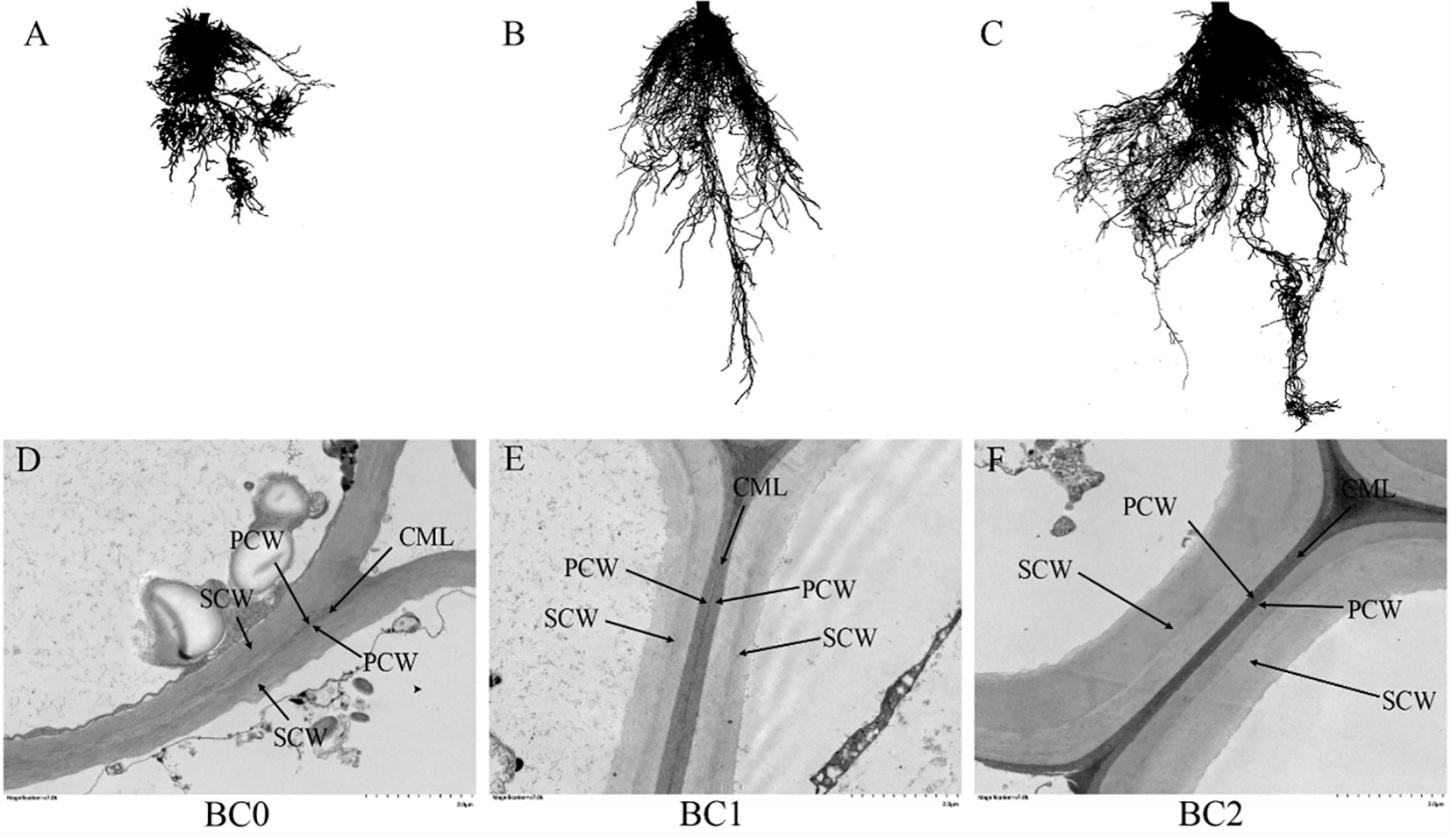
The root morphology and cell ultrastructure of root tips under different biochar treatments. **(A, D)** no biochar addition; **(B, E)** 5% biochar addition in copper-contaminated soil; **(C, F)** 10% biochar addition in copper-contaminated soil. CML, compound middle lamella; PCW, primary cell wall; SCW, secondary cell wall.

The root morphology and cell wall ultrastructure of root tips are shown in **Figure 6**. It revealed that the root growth inhibition caused by excessive copper stress was alleviated by the addition of biochar (**Figures 6A–C**). Meanwhile, the TEM observation revealed that copper stress negatively altered the cell wall ultrastructure, and the addition of biochar in Cu-contaminated soil increased the mechanical strength of the cell wall, explicitly increasing the thickness of the secondary cell wall with clear layers (**Figures 6D–F**). The biochar addition significantly increased the root morphological traits and activity (**Table 2**). The TRL in BC1 and BC2 were 1.5-fold and 3.6-fold than that in BC0. The RSA increased by 41% in BC1 and 133% in BC2, respectively, compared to BC0. The RV, RD, and RT increased by 85%, 70%, and 389%, respectively, in the pots where 10% biochar was added. A similar trend was found for root activity. In contrast to BC0, the BC1 treatment increased the root activity by 40%, and the BC2 treatment vastly improved the root activity by 113%.

**Table 2:** The effects of biochar application on root agronomic traits and root activity under Cu-contaminated soil.

Treatment	TRL	RSA	RV	RD	RT	Root activity
	cm	cm^2^	cm^3^	cm	-	-
BC0	95.5 ± 13.8c	35.1 ± 5.8b	2.7 ± 0.3c	0.62 ± 0.1b	142.3± 18.3c	0.15 ± 0.01c
BC1	143.9 ± 14.4b	49.6 ± 4.9b	3.9 ± 0.7b	0.98 ± 0.1a	308.7 ± 28.7b	0.21 ± 0.02b
BC2	371.8 ± 10.7a	81.9 ± 5.4a	5.0 ± 0.6a	1.06 ± 0.1a	696.3 ± 42.1a	0.33 ± 0.04a

The values are expressed as mean ± standard error for n = 3 replications. Different letters indicate statistically significant differences at P ≤ 0.05. BC0, BC1, and BC2 indicate applying 0, 5%, and 10% biochar in the Cu-contaminated soil, respectively. TRL, total root length; RSA, root surface area; RV, root volume; RD, root diameter; RT, root tips.

### 3.5 Remediation efficiency, yield production and soil amelioration

**Figure 7:**
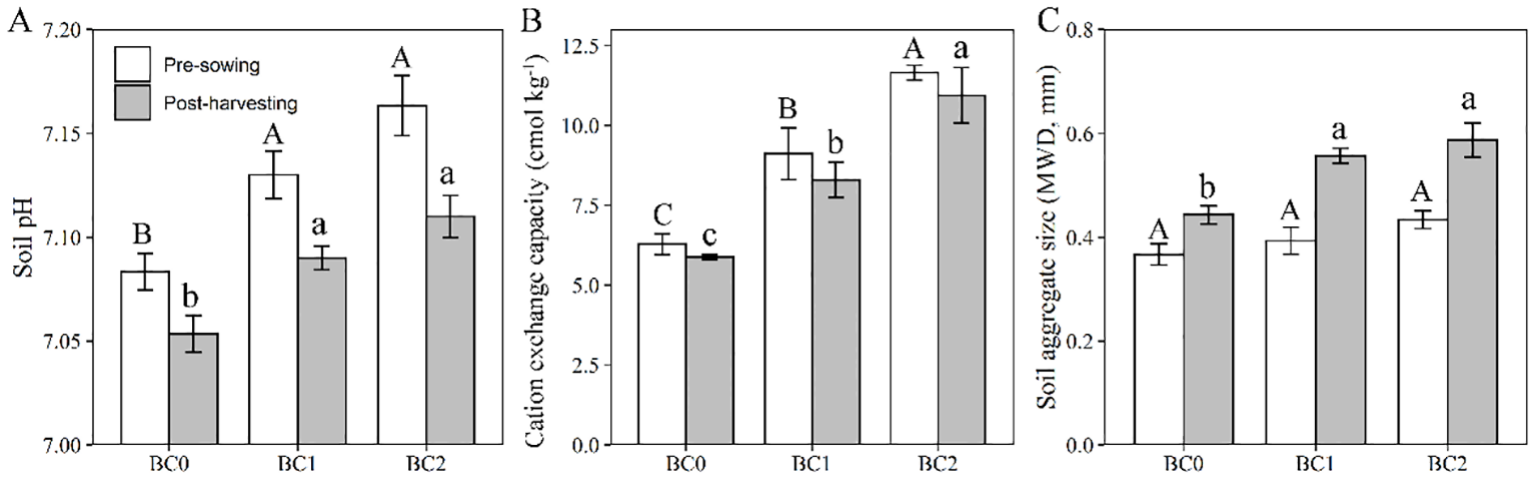
The effects of biochar application rates and rapeseed cultivation on soil properties. **(A)** soil pH; **(B)** soil cation exchange capacity; **(C)** soil aggregate size. The values are expressed as mean ± standard error for n = 3 replications. Different letters indicate statistically significant differences at *P* ≤ 0.05; Uppercase letters to the pre-sowing stage and lowercase letters to the past-harvesting stage.

The effects of biochar application and rapeseed cultivation on the physicochemical properties of Cu-contaminated soil are shown in **Figure 7**. After two months’ equilibrium with biochar added in Cu-contaminated soil at the pre-sowing stage, the soil pH significantly elevated from 7.08 to 7.13 at 5% biochar application, and to 7.16 at 10% biochar application (**Figure 7A**). ANOVA analysis revealed that soil pH showed a significant decrease after rapeseed cultivation in contrast to the pre-sowing stage (*F* = 24, *P* < 0.01). The soil cation exchange capacity significantly increased with the biochar application (**Figure 7B**) and showed a slightly declined trend after rapeseed cultivation with no significance (*F* = 1.99, *P* = 0.18). The soil aggregate size at the pre-sowing stage slightly increased with the increasing of biochar addition rates in Cu-contaminated soil, and significant improvement relative to BC0 was observed at the post-harvesting stage (**Figure 7C**). Meanwhile, the rapeseed cultivation significantly increased the soil aggregate size (*F* = 24, *P* < 0.01).

The biochar remediation was evaluated as the reduction of bioavailable Cu in soil with biochar addition relative to BC0 after two months’ equilibrium with biochar application. The BC1 treatment decreased the bioavailable Cu by 14.5 mg kg^-1^ with a 10.0% remediation efficiency, and the BC2 treatment showed a 12.3% remediation efficiency (**Table 3**). The reduction of bioavailable Cu at the post-harvesting stage in relative to the pre-sowing stage was ascribed to rapeseed phytoremediation. The bioavailable Cu decreased by 5.0 mg kg^-1^ in BC0 treatment, with a 4.9% phytoremediation efficiency. The 5% and 10% biochar addition in Cu-contaminated soil showed 9.0% and 13.6% phytoremediation efficiency, respectively (**Table 3**). The addition of biochar in Cu-contaminated soil significantly improved the seed yield of rapeseed. Our results showed that 5% and 10% biochar addition increased the seed yield by 48% and 106%, respectively (**Table 3**).

**Table 3:** The effects of biochar application treatments on the soil bioavailable Cu concentration (mg kg^-1^), remediation efficiency (%) and rapeseed yield (g plant^-1^).

Treatment	Bioavailable Cu in soil		Remediation efficiency		Seed yield
	Pre-sowing	Post-harvesting	Biochar	Rapeseed	
BC0	99.8 ± 2.30a	94.8 ± 1.49a	–	4.9	6.52 ± 0.18c
BC1	85.3 ± 1.55b	77.6 ± 1.40b	10.0	9.0	9.67 ± 0.20b
BC2	78.8 ± 1.16c	68.1 ± 2.41c	12.3	13.6	13.48 ± 0.84a

The values are expressed as mean ± standard error for n = 3 replications and “-” indicate value not available. Different letters indicate statistically significant differences at P < 0.05.

## 4 Discussion

Combining multiple remediation technologies offers a promising approach to enhance the efficiency of heavy metal-contaminated soil remediation (Abbas et al., 2020; Zhang et al., 2020). The key findings from the present study revealed that incorporating biochar with rapeseed, a heavy metal accumulator, improves the remediation efficiency of copper-contaminated soils and increases rapeseed yield. A 10% biochar addition to copper-contaminated soil reduced copper bioavailability by 12.3% through complexation and precipitation, while rapeseed further reduced available copper by 13.6% via root extraction. These findings align with previous studies showing synergistic effects between biochar and phytoremediation. For instance, 5% cornstalk biochar in Cd-contaminated soil decreased Cd extractability by 30% and enhanced Cd uptake in *Phytolacca americana* L. roots (Zhang et al., 2020). Similarly, 10% manure biochar with B*rassica napus* growth significantly reduced As, Cu, Co, Cr, Se, and Pb in mining soil (Gasco et al., 2019). Remediation mechanisms consist of two fundamental principles: (i) to remove contaminations from polluted sites and (ii) to transform them into harmless forms (Wei et al., 2008). A significant concern to highlight is that the remediation system in this study primarily focuses on the immobilization of copper, as approximately 50% of the heavy metals are sequestered in the roots during the phytoremediation process. This presents a challenge as these metals cannot be removed as readily as in traditional hydroponic conditions used in rhizofiltration processes. To effectively remove heavy metals from the soil, it is crucial to enhance harvesting methods for extracting roots from the soil in practical applications.

### 4.1 Biochar’s role in alleviating copper toxicity in rapeseed

Biochar application improved the rapeseed growth rate by alleviating copper toxicity. Overproduction of ROS is considered the primary causative agent of tissue injury after exposure of plants to excessive copper stress (Ali et al., 2006; Hartley‐Whitaker et al., 2001). The decrease of copper toxicity and oxidative stress was observed by reducing MDA, O^2-^ and H_2_O_2_ contents in the leaf and root of rapeseed with increasing biochar application. Histochemical analyses with 3,3′-diaminobenzidine (DAB) and nitro blue tetrazolium (NBT) further confirmed that the generation of O_2_^-^ and H_2_O_2_ was inhibited with the increase of biochar application. This coincided with the decreased levels of enzymatic and non-enzymatic antioxidants (SOD, POD, CAT, APX, and Pro) under biochar addition. Excess copper in soil reduced the photosynthesis efficiency and ultimately hampered plant growth and development (Jin et al., 2021; Vinit-Dunand et al., 2002). High Cu^2+^ concentrations at the cellular level caused the substitution of the central Mg^2+^ in the chlorophyll molecules and lipid peroxidation of thylakoid membranes, interfered with electron transport in the photochemical reactions of PSII, leading to inhibition of photosynthesis (Küpper et al., 2003; Perales-Vela et al., 2007; Tiecher et al., 2017). The addition of 10% rice straw biochar in Cu-contaminated soil raised the rapeseed biomass by 2.7 times relative to control. Plant biomass and heavy metal concentration in plant organs are vital factors in determining phytoremediation efficiency (Zhang et al., 2021). Our results showed that increasing biochar addition from 0 to 10% application rates decreased the copper concentrations in rapeseed tissues. However, it is important to note that the markable increase in biomass offset the negative effect of the decrease in copper concentration, and 10% application rates of biochar maximized the absorption amount of copper in contaminated soil.

### 4.2 Biochar and rapeseed synergy in copper remediation

The bioavailable copper in contaminated soil decreased with increasing biochar application due to processes such as adsorption, precipitation, or complexation. Specifically, the addition of 5% and 10% biochar reduced bioavailable Cu concentrations by 10.0% and 12.3% compared to the control, respectively. Biochar, characterized by its carbon-rich organic composition, microporous structure, alkaline pH, and high cation exchange capacity, exhibits a strong affinity for heavy metals (Shi et al., 2022; Wang and Wang, 2019). In this study, rice straw biochar with an alkaline pH of 8.15 facilitated the precipitation of available copper in copper-contaminated soil, likely forming copper hydroxide. Incorporating 10% biochar enhanced the soil’s cation exchange capacity from 6.28 to 11.29 prior to sowing. FTIR analysis revealed prominent peaks at 1096 cm^-1^, 1616 cm^-1^, and 3447 cm^-1^ in rice straw biochar, indicating significant concentrations of C–O, C=O, and –OH functional groups. The biochar’s effectiveness in binding heavy metals can be largely attributed to these oxygen-containing functional groups on its surface (Fan et al., 2018; O'Connor et al., 2018).

Biochar application could enhance the phytoremediation of rapeseed under severe Cu-contaminated soil. Our results showed that the rapeseed growth under the non-biochar applicate condition reclaimed 4.9% copper from the Cu-contaminated soil. The 5% and 10% biochar addition in the Cu-contaminated soil improved the phytoremediation efficiency of rapeseed to 9.0% and 13.6%, respectively. One mechanism of how biochar promoted copper accumulation of rapeseed could be explained by the increase of hemicellulose and pectin content in rapeseed’s root and shoot cell wall. This finding aligns with results observed in rice, where an increase in pectin and hemicellulose content, coupled with a decrease in cellulose, has been shown to enhance cadmium accumulation in the root cell wall (Xiong et al., 2009). The plant cell wall is implicated as a primary barrier to prevent heavy metal ions from entering the plant cytoplasm (Jia et al., 2019). Hemicellulose and pectin are rich in various negatively charged organic functional groups, and elevating their contents improved the plant’s capacity to sequestrate heavy metals in cell walls (Cao et al., 2024; Konno et al., 2010). Previous literature has reported that 75% of the Cr accumulated in the root was contained in the cell wall, and 63% of the Cr accumulated in the shoot was found in the vacuoles and cell walls (Wu et al., 2013). The root was the main plant organ for copper accumulation for rapeseed and adding 5% biochar increased the ratio of copper amount in root to whole plant from 34% to 54%. The 10% biochar application further increased this value up to 58%. This changing pattern was conforming to the increased ratio of root to shoot with the elevated levels of biochar addition in Cu-contaminated soil.

### 4.3 Soil amelioration and economic implications

Monitoring and evaluating the physiochemical characteristics of soil and yield production of rapeseed was a crucial part of the joint remediation project in the present study. The soil pH slightly decreased, and the soil aggregate size notably increased at post-rapeseed harvesting compared to pre-sowing, implying that rapeseed roots secreted several substances to ameliorate soil physical properties. Heavy metal stress stimulates the biosynthesis of organic acids and releases them from roots (Panchal et al., 2021; Yizhu et al., 2020). Similarly, previous literature reported that *Agrostis capillaris* L. and *Lupinus albus* L. exhibited rhizospheric acidification in the phytoremediation process with biochar application (Houben and Sonnet, 2015). Soil cation exchange capacity slightly decreases after rapeseed cultivation, which could be ascribed to decreased soil pH. The cation exchange capacity was strongly pH-dependent, and a lowering of soil pH resulted in a decline in surface negative charge, thus lowering the CEC (Mehta et al., 2003; Silber et al., 2010). Increasing biochar application to 10% (w/w) doubled the seed yield production of rapeseed relative to the non-biochar application. In addition, rapeseed is commercially cultivated for vegetable oil extracted from the seed; the copper concentration in seed showed a limited accumulation, which is lower than the tolerance limit of copper in food provided by standard Chinese GB15199-94 (Lin et al., 2014). Thus, applying rapeseed and biochar synergy to remediate Cu-contaminated soil could produce ecological and economic benefits.

## 5 Conclusion

In our experiment, adding 10% straw rice biochar to Cu-contaminated soil significantly reduced the bioavailable copper concentration and promoted the growth of rapeseed. Increasing biochar application reduced O_2_^-^, H_2_O_2_, and MDA contents in the leaves and roots of rapeseed, mitigating the negative impacts of excessive copper stress. Higher doses of biochar improved both the remediation efficiency of biochar and the phytoremediation efficiency of rapeseed cultivation. Additionally, rapeseed cultivation with biochar application significantly increased rapeseed yield and improved soil physicochemical properties. The copper concentration in seeds was below the tolerance limit set by Chinese standard GB15199-94. This study highlights the potential of phytoremediation-biochar synergy for remediating Cu-contaminated soil, offering ecological benefits and producing edible or biofuel oil for economic gains. However, further research is needed to evaluate the long-term and large-scale effectiveness of biochar application in rapeseed cultivation under field conditions.

## Data Availability

The original contributions presented in the study are included in the article/supplementary material. Further inquiries can be directed to the corresponding author.
